# Thermomagnetic Resonance Effect of the Extremely Low Frequency Electromagnetic Field on Three-Dimensional Cancer Models

**DOI:** 10.3390/ijms23147955

**Published:** 2022-07-19

**Authors:** Loredana Bergandi, Umberto Lucia, Giulia Grisolia, Iris Chiara Salaroglio, Iacopo Gesmundo, Riccarda Granata, Romano Borchiellini, Antonio Ponzetto, Francesca Silvagno

**Affiliations:** 1 Department of Oncology, University of Torino, Via Santena 5 bis, 10126 Torino, Italy; loredana.bergandi@unito.it (L.B.); irischiara.salaroglio@unito.it (I.C.S.); 2 Department of Energy, Politecnico di Torino, Corso Duca degli Abruzzi 24, 10129 Torino, Italy; umberto.lucia@polito.it (U.L.); giulia.grisolia@polito.it (G.G.); romano.borchiellini@polito.it (R.B.); 3 Department of Medical Sciences, University of Torino, Corso A.M. Dogliotti 14, 10126 Torino, Italy; iacopo.gesmundo@unito.it (I.G.); riccarda.granata@unito.it (R.G.); antonio.ponzetto@unito.it (A.P.)

**Keywords:** extremely low frequency electromagnetic field, cancer spheroids, thermodynamic approach, human pancreatic cancer, human glioblastoma, human breast cancer, proliferation, mitochondrial respiration, electron transport chain, uncoupling proteins

## Abstract

In our recent studies, we have developed a thermodynamic biochemical model able to select the resonant frequency of an extremely low frequency electromagnetic field (ELF-EMF) specifically affecting different types of cancer, and we have demonstrated its effects in vitro. In this work, we investigate the cellular response to the ELF electromagnetic wave in three-dimensional (3D) culture models, which mimic the features of tumors in vivo. Cell membrane was modelled as a resistor–capacitor circuit and the specific thermal resonant frequency was calculated and tested on two-dimensional (2D) and three-dimensional (3D) cell cultures of human pancreatic cancer, glioblastoma and breast cancer. Cell proliferation and the transcription of respiratory chain and adenosine triphosphate synthase subunits, as well as uncoupling proteins, were assessed. For the first time, we demonstrate that an ELF-EMF hampers growth and potentiates both the coupled and uncoupled respiration of all analyzed models. Interestingly, the metabolic shift was evident even in the 3D aggregates, making this approach particularly valuable and promising for future application in vivo, in aggressive cancer tissues characterized by resistance to treatments.

## 1. Introduction

The beneficial effects of the extremely low frequency electromagnetic field (ELF-EMF) were investigated in recent studies both in vitro [1] and in vivo [2], in an attempt to exploit the electromagnetism for medical applications [3] and in cancer therapy [4,5,6]. Indeed, in the last decades several studies revealed not only that exposure to an ELF-EMF does not induce DNA damage (as ionizing radiation do), but also that it has the potential to affect cellular processes, such as proliferation, differentiation and apoptosis, which can be exploited in cancer treatments [7]. Interestingly, we show that when cancer cells were stimulated with an ELF-EMF, we were able to select the appropriate frequency driving a global response, evaluated as entropy variation affecting cancer growth and motility through a metabolic shift [8,9,10]. In fact, we described the molecular basis of the growth inhibition that relies on the mitochondrial metabolic switch, necessary to control ion fluxes and deleterious to cancer cell proliferation [8].

The search in the oncological field for new therapeutic strategies is particularly active, since standard antineoplastic treatments based on surgery, chemotherapeutic drugs and/or radiotherapy have potentially detrimental secondary effects and, often, non-specificity in cytotoxic action [11]. Moreover, certain patients have a low survival rate because of potential chemotherapy resistance to various highly aggressive tumors. This is the case in small-cell lung cancer, triple-negative breast cancer, pancreatic ductal adenocarcinoma, glioblastoma, metastatic melanoma and advanced ovarian cancer, characterized by a highly invasive phenotype and lack of biomarker expression for target therapies [12]. Pancreatic ductal adenocarcinoma (PDAC) is one of the most aggressive cancers, with poor prognosis and a five-year survival rate of less than 8% because of the absence of early symptoms, late diagnosis and the resistance to radio- and chemotherapy [13]. The existence of a dense tumor microenvironment (TME) may be the main reason why therapies specifically targeting only cancer-associated molecular pathways have not given satisfactory results [14]. Indeed, the TME of PDAC is characterized by abundant stroma, associated with areas of hypoxia with a significant reduction in oxygen tissue levels and acidic extracellular pH, a deficient blood supply, a reprogrammed metabolism and elevated immunosuppression [15]. Glioblastoma (GBM), the most common and aggressive central nervous system (CNS) malignancy with a 5-year overall survival of 5.6%, represents 47.7% of all primary malignant tumors of the CNS [16]. One of the constitutive features of these tumors is their heterogeneity and histopathological hallmarks involving nuclear atypia, necrosis, mitosis and microvascular proliferation [17]. Moreover, its aggressiveness is aggravated by the side effects of surgery, radiation and chemotherapeutic agents [18]. Breast cancer (BC) is a heterogeneous disease comprised of distinct biological subtypes, and cells with differing receptor status may coexist in the same tumor [19]. Currently, strategies for the treatment of BC depend on the tumor subtype, and the selected treatments are directed to specific targets that are functionally altered in each subtype [20]. Even if therapies improvement is a milestone in BC therapy, many BC patients still experience poor drug response and tumor recurrence in clinical observation, exhibiting intrinsic drug-resistance or acquiring resistance to anticancer drugs [21,22]. Indeed, about 30% of patients with metastatic disease have objective regression of tumor with initial treatment, while another 20% have prolonged stable disease [23].

The research in vitro has exploited the possibility of investigating the intercellular communication in spheroid shaped growing cells. Multicellular spheroids (MCSs) developed in three-dimensional (3D) culture are believed to more closely mimic solid tumors, with respect to cell–cell interactions, hypoxia, drug penetration and nutrition gradients [24], which are irreproducible in a conventional two-dimensional (2D) cell culture [25]. Thus, in the oncology field, 3D suspension culture has emerged as a tool for capturing critical molecular and functional properties of cancer, because of the different biological characteristics of MCSs in 3D suspension culture and parental cells grown in the conventional 2D-adherent culture [26,27].

Up to now, the effects of an ELF-EMF applied to 3D cancer cellular cultures were not investigated. In fact, our previous thermodynamic analysis on cancer was validated on monolayers of cancer cells. As published in our most recent studies [28,29,30,31], life can be modelled as a thermodynamic biophysical process, based on the thermodynamic principle of the maximum conversion of available energy [30]. From a thermodynamic viewpoint, a cell is a system characterized by energy and matter fluxes through the cell membrane [32]. The ELF electromagnetic wave induces a homeostatic response that is supported by a shift in cellular energy conversion and a metabolic modulation, triggering an increase in entropy coupled to a decrease in cell growth. We have used the thermodynamic approach to build a mathematical model able to predict the characteristics of the electromagnetic wave able to stimulate the changes of entropy in the cell-environment system [10]. Moreover, we further developed the thermodynamic formulation by introducing the thermal exchange analysis and by describing the resonant effects triggered by the exposure of cells to an ELF-EMF [9]. The theoretical analysis has been verified on many bidimensional models of cancer, so far [8,9,10].

Based on our thermodynamic approach, the stimulation of a 3D mass with the specific ELF-EMF should influence cell behavior similarly to what is previously demonstrated on cancer cell monolayers. In the present work, we compared the effects of the exposure on both adherent cells and spheroids derived from the same tumor type to investigate the cellular response to the ELF electromagnetic wave in a 3D culture model. This study is a fundamental further step in our thermodynamic approach, by which we integrate previous considerations in a novel formal analysis, revealing the effects of an ELF-EMF on the metabolism of a 3D structure similar to an in vivo tumor mass [33]. First, we evaluated by a thermodynamic approach the variation of ionic fluxes and the heat dissipation that can be triggered in a cancer mass by a thermal resonant frequency of the ELF-EMF. Then, by our thermodynamic model [8,9,10] we calculated the specific frequencies for three models of human cancer, available both as monolayer and 3D cultures, and most importantly, we investigated the biological impact of the treatment in terms of cell growth and mitochondrial activity.

## 2. Results

### 2.1. The Thermodynamic Approach Allows to Calculate the Thermal Resonant Frequency of ELF-EMF Specific for Two-Dimensional and Tri-Dimensional Cancer Models

Cellular biochemical reactions perform work (replication, transcription and translation, and maintenance of gradients) by using inflowing metabolites, and releasing heat flow towards cell environment.

Therefore, a thermodynamic approach (the engineering thermodynamic bases in biophysics are summarized in the Appendix A Section) allows us to analyze the cell by introducing the black box approximation for the cell system, and by considering the genetic regulation as a control tool of the cellular processes, because the genetic regulation cannot affect the thermodynamic balance of the cellular system. Cells exchange heat with their environment by convection with the fluids around them, and the energy balance of this process brings to [34]:

(1) $$\left\{ \begin{array}{l} \dot{Q}=\alpha A\left(T_{cell}-T_{0}\right)=\alpha \frac{V}{\langle r\rangle }\left(T_{cell}-T_{0}\right)\\ \dot{Q}=\rho_{cell}\;V\;c_{cell}\;\frac{dT_{cell}}{dt} \end{array}\right.$$

where $\dot{Q}$ is the heat power flux, *ρ_cell_* is the mass density of the cell, *V* is its volume, *c_cell_* is its specific heat, *T_cell_* is the cell temperature, *α* is the convection coefficient, *A = V*/<*r*> is the surface area of the cell, which varies during the phases of the cellular development, <*r*> = *V*/*A* is the volume-area ratio, that is a characteristic parameter of the heat exchange through the cell membrane and (*T_cell_* − *T*_0_) is the temperature difference between the cell temperature and the environmental temperature (*T*_0_). Consequently, the Equation (1) brings to the differential equation:

(2) $$\frac{1}{T_{cell}-T_{0}}\;\frac{d\left(T_{cell}-T_{0}\right)}{dt}=\frac{\alpha }{\rho_{cell}c_{cell}}\frac{1}{\langle r\rangle }$$

which is well known in heat transfer theory. Usually, when the Biot number result lower is than 0.1, this equation can be solved by considering the lumped parameter model, by introducing the characteristic time *τ*, defined as follows [34]:

(3) $$\frac{\alpha }{\rho_{cell}c_{cell}}\frac{1}{\langle r\rangle }=\frac{1}{\tau }$$

The cell membrane is usually modelled as a resistor–capacitor circuit (*RC* circuit) [35], where the current across the resistor of resistance *R* is evaluated during the transient process on the capacitor of capacity *C* [36]:

(4) $$I\left(t\right)=\frac{\Delta \phi }{R}\;e^{-t/\tau_{circ}}$$

where *I*(*t*) is the electric current, *t* is the time, ∆*ϕ* is the electric potential applied to the circuit, *R* is the electric resistance and *τ_circ_* = *RC* is the electric characteristic time of the circuit. When a harmonic electromagnetic wave with a resonant frequency *ν* ~ *τ_circ_*^−1^ = (*RC*)^−1^ [36] interacts with the circuit, a heat power is dissipated in the resistor by the Joule effect, as follows:

(5) $$\dot{Q}\left(t\right)=\frac{R\;\phi_{M}^{2}}{\sqrt{R^{2}+\tau_{circ}^{2}{\left(2\pi C\right)}^{-2}}}\;\sin^{2}\left(\frac{2\pi }{\tau_{circ}}t\right)$$

where *ϕ_M_* is the maximum value of the electric potential of the electromagnetic wave.

In accordance with the *RC* analogy for the cell membrane, a characteristic time (Equation (3) can also be considered [37] in the thermal interaction between cells and environment, such that *τ_circ_* = *τ*. Consequently, an electromagnetic wave could generate a resonant effect, with a frequency inversely proportional to this time [31]. Therefore, the characteristic time *τ* in Equation (3) is evaluated and an electromagnetic wave is inflowed onto cells, at the frequency 1/*τ*. The released heat flow results:

(6) $$\dot{Q}=\frac{Q}{\tau }=T_{0}\frac{\Delta S_{cell}}{\tau }$$

where *Q* is the heat wasted by the cell towards its environment, *T*_0_ is the environmental temperature and ∆*S_cell_* is the entropy variation of the cell. Now, considering the definition of the Gibbs free energy *G* [38]:

(7) $$\Delta G=\Delta H-\dot{Q}\;\tau$$

where *H* is the enthalpy, and *T*_0_ is the environmental temperature, and considering the relation between the Gibbs free energy, the membrane electric potential and the pH [39]:

(8) $$\Delta G=\Delta \phi -2.3\frac{R_{u}T_{0}}{F}\Delta \mathrm{pH}$$

where *φ* is the cell membrane electric potential, *R_u_* is the universal gas constant, *F* is the Faraday constant and pH is the potential of hydrogen, the application of an electromagnetic wave, at the characteristic frequency, generates a change in the membrane electric potential:

(9) $$\Delta \phi =\Delta H-\dot{Q}\;\tau +2.3\frac{R_{u}T_{0}}{F}\Delta \mathrm{pH}$$

with a related variation in the ion concentration at the membrane forced in accordance with the relation [31,37]:

(10) $$c_{out}=c_{in}\exp\left(\frac{\Delta \phi }{R_{u}T_{0}}\right)$$

where *c_out_* and *c_in_* are the concentrations of any ion species outside and inside of the cell membrane. The previous relation (8) is the well-known Nernst equation in electrochemistry. Here, it is used to link the membrane electric potential to the Gibbs free energy variation, in order to consider the thermodynamic effects related to the entropy variation caused by the external fields. Indeed, external fields generate a resonant effect on the thermal fluxes which are related to the entropy flux. In order to understand their effect on the cell behavior it is fundamental to obtain an analytical relation between this entropy flux and the membrane electric potential: this is the result obtained by introducing just the Nernst equation.

Based on cell morphology, it is possible to calculate the characteristic resonant frequency of the ELF-EMF, as previously demonstrated [8,9,10]. At this frequency, the cell, whose membrane is modelled as an *RC* circuit, generates ion fluxes (current) and dissipates heat (Equation (6)). We can thus calculate the electric potential (Equation (9)) and we conclude that, at the thermal resonant frequency, a variation of ionic fluxes is induced (Equation (10)), from which the cell must defend itself by a principle of homeostasis. For this purpose, it is forced to increase energy production and restore ion fluxes through adenosine triphosphate (ATP)-dependent mechanisms. Since heat is dissipated in a liquid extracellular matrix, and the multicellular aggregate as a whole is isothermal, the considerations and laws applied to the single cell are also referable to the entire three-dimensional tumor mass, which is built as a spheroid in our experimental model.

We previously demonstrated that, based on our mathematical model, we could select the frequencies specific for each cancer cell type, showing that the ELF-EMF arrested cell growth and rewired cell metabolism [8,9,10]. In this study, based on the proposed theoretical model, we calculated the frequencies of the ELF-EMF able to affect the growth and metabolism of three different models of cancer, growing as tridimensional masses, and compared the impact on the irradiated 3D models with the expected response of the same cell type growing as a two-dimensional adherent population. Relative to their size and morphology, for each cell type we calculated the specific optimal thermomagnetic resonant frequency, which resulted in 6 Hz for MCF-7 human breast cancer cells, in both the adherent and 3D model, 4 Hz for the 01627 human GBM adherent cells and 6 Hz for the 01627 human GBM spheroid culture. The optimal frequency for PANC-1 human pancreatic cancer cells grown as 3D masses was 3 Hz, whereas the adherent PANC-1 cells demonstrated a noticeable heterogeneity in shape and dimensions. Therefore, we estimated four different frequencies able to affect distinct subpopulations, and these frequencies of 3, 6, 10 and 14 Hz were tested separately. The six cancer models are shown in Figure S1.

### 2.2. ELF-EMF Exposure Inhibited Cell Growth of Human Pancreatic Epithelioid Carcinoma Adherent Cells and Cell-Derived Spheroids

**Figure 1:**
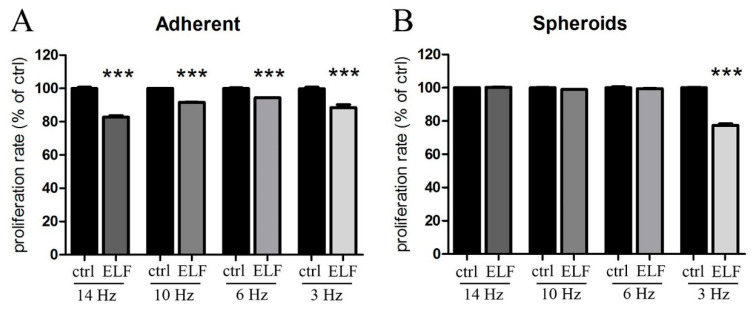
The exposure to the specific electromagnetic wave inhibits the proliferation of adherent (**A**) and spheroids (3 Hz) (**B**) human pancreatic epithelioid carcinoma (PANC-1) cell line. After two days of growth in standard condition (ctrl) or in presence of ELF-EMF at the indicated frequencies (ELF), adherent and spheroids of PANC-1 cells were subjected to BrdU assay. The values of the treated cells are expressed as the percentage of their respective controls (ctrl). The data represent the means ± SEM of three independent experiments. *** *p* < 0.001 compared to the control.

After 2 days of exposure to specific frequencies of the ELF-EMF, bromodeoxyuridine/5-bromo-2′-deoxyuridine (BrdU) assay of control and treated cells was performed to evaluate the inhibition of cellular proliferation. Figure 1A shows that PANC-1 adherent cells were sensitive to all the calculated different frequencies (14, 10, 6 and 3 Hz), due to the heterogeneity of the cellular population (Figure S1). The most effective was 14 Hz, corresponding to the frequency calculated as optimal for the smallest round shaped cells, which probably are the most actively dividing cells. Interestingly, only the defined frequency of 3 Hz was efficiently decreasing the growth of the PANC-1 spheroids (Figure 1B), as the cell morphology was unique (Figure S1). These results demonstrated the specificity of the applied ELF-EMF and confirmed the effect of the specific exposure also on the 3D model.

### 2.3. Mitochondrial Respiration Is Increased by ELF-EMF Exposure in Both Human Pancreatic Epithelioid Carcinoma Adherent Cells and Cell-Derived Spheroids

**Figure 2:**
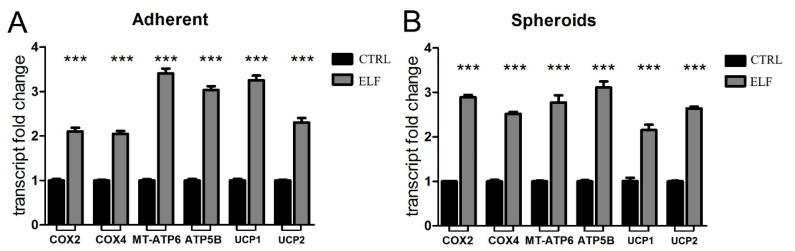
The exposure to the specific electromagnetic wave enhances the transcription of the components of mitochondrial respiration. In adherent PANC-1 cells (**A**) and in PANC-1 spheroids (**B**) the real-time PCR analysis quantified the transcripts of cytochrome *c* oxidase subunit 2 (COX2) and subunit 4 (COX4), ATP synthase subunits (MT-ATP6 and ATP5B), and uncoupling proteins UCP1 and UCP 2. The cells were treated for two days at the specific frequencies: 14 Hz for adherent cells and 3 Hz for spheroids. Fold changes versus control are plotted on the graphs. All data represent the mean ± SEM of three independent experiments. *** *p* < 0.001 compared to control.

It has been previously reported that the ELF-EMF influences the mitochondrial activity in different cancer cell types in vitro [8,9]. To evaluate the impact of the ELF-EMF on the coupled and uncoupled respiratory activity, we assessed the mRNA levels of several proteins involved in energy production, such as mitochondrial respiratory complexes, ATP synthase and uncoupling proteins, in both adherent and 3D models of PANC-1 cells. The exposure to 14 Hz, chosen as the most effective frequency in decreasing pancreatic adherent cell proliferation, and the treatment of PANC-1 spheroids with a 3 Hz EMF, enhanced the mitochondrial activity in each model (Figure 2A,B). In fact, after 2 days of ELF-EMF exposure at these defined frequencies, we observed a significant increase in mRNA levels of two subunits of complex IV: cytochrome *c* oxidase subunit 2 (COX2) and subunit 4 (COX4), whose transcripts are of mitochondrial (the former) and nuclear (the latter) origin. In addition, two subunits of ATP synthase were upregulated in their nuclear (ATP5B) and mitochondrial transcription (MT-ATP6), as well as the uncoupling proteins (UCPs) UCP1 and UCP2. All together, these results confirm the impact of the selective ELF-EMF frequency on mitochondrial metabolism of adherent cells. Of note, the same effect was obtained when cells aggregated in a 3D mass, a condition that mimics their interactions in vivo.

**Figure 3:**
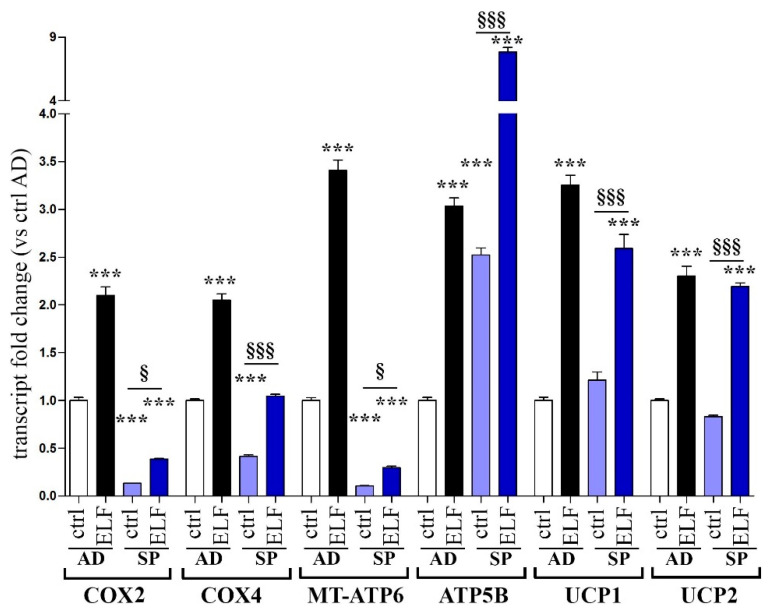
Comparison of transcript levels in adherent or 3D PANC-1 cell cultures. Adherent (AD) or spheroids (SP) cells were left untreated (ctrl) or exposed to ELF-EMF (ELF) for two days. The transcripts of cytochrome *c* oxidase subunit 2 (COX2) and subunit 4 (COX4), ATP synthase subunits (MT-ATP6 and ATP5B) and uncoupling proteins UCP1 and UCP 2 were quantified by real time PCR. Fold changes versus control adherent cells (white bars) are plotted on the graphs. Data represent the mean ± SEM of three independent experiments. *** *p* < 0.001 compared to control AD; § *p* < 0.05 compared to control SP; §§§ *p* < 0.001 compared to control SP.

Furthermore, we compared the levels of the respiratory chain transcripts in adherent cells and spheroids. Figure 3 shows that in untreated cells, the respiratory complexes COX2 and COX4 were less expressed in the 3D model, compared with adherent cells, although the exposure to the ELF-EMF increased the transcription in both models. Similarly, the mitochondrial transcription of ATP synthase subunit MT-ATP6 was downregulated in untreated spheroids but sensitive to ELF-EMF stimulation. On the opposite, the subunit ATP5B was upregulated in spheroids, whereas the transcript levels of UCPs were not changed by 3D cell aggregation. Despite these differences, after ELF-EMF exposure we still observed a significant induction of mitochondrial respiratory activity in treated spheroids, with a fold increase similar to that measured in adherent cells.

### 2.4. ELF-EMF Exposure Inhibited the Growth of Adherent Cells and Cell-Derived Spheroids and Increased Mitochondrial Activity of Human Glioblastoma Cells

**Figure 4:**
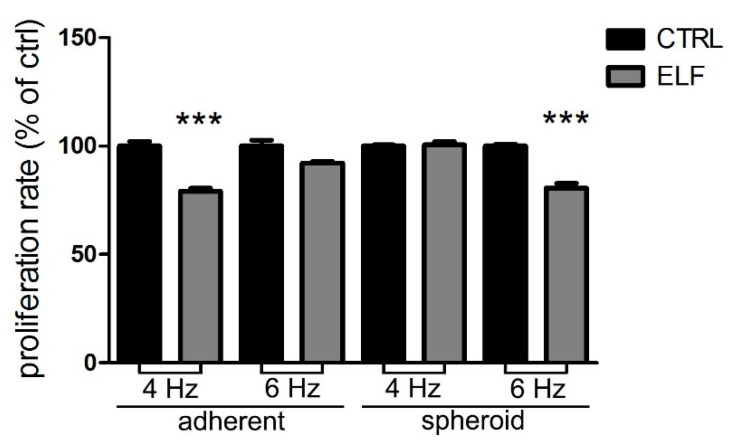
The exposure to the specific electromagnetic wave inhibits the proliferation of cells cultured as adherent cells and neurospheres of primary human glioblastoma multiform (GBM). After two days of growth in standard condition (ctrl) or in presence of ELF-EMF at the indicated frequencies, adherent and neurospheres of GBM cells were subjected to BrdU incorporation. The values of the treated cells are expressed as the percentage of their respective controls (ctrl). Data represent the means ± SEM of three independent experiments. *** *p* < 0.001 compared to the control.

Our results were subsequently confirmed in a different aggressive type of solid tumor, the primary 01627 human glioblastoma multiforme, which was grown in both the differentiated component as adherent cells and in the staminal component as neurospheres. In this human glioblastoma model, at the specific frequency of 4 Hz for adherent glioblastoma cells, and 6 Hz for neurospheres, ELF-EMF exposure slowed down cell growth (Figure 4), whereas the nonspecific frequency had no effect on cell proliferation.

**Figure 5:**
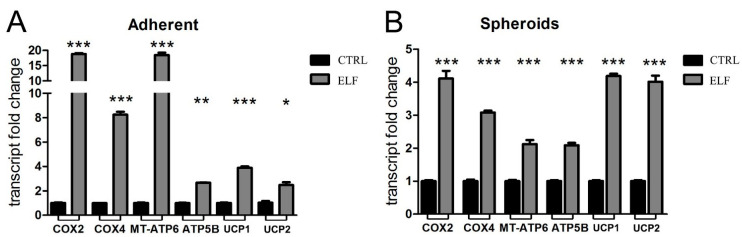
The exposure to the specific electromagnetic wave enhances the expression of the components of mitochondrial respiration. Cells cultured as adherent cells (**A**) and neurospheres (**B**) of primary human GBM were treated for two days with ELF-EMF (ELF) at the specific frequencies: 4 Hz for adherent cells and 6 Hz for neurospheres. The transcripts of cytochrome *c* oxidase subunit 2 (COX2) and subunit 4 (COX4), ATP synthase subunits (MT-ATP6 and ATP5B) and uncoupling proteins UCP1 and UCP 2 were quantified by real time PCR. Fold changes versus control (CTRL) are plotted on the graphs. All data represent the mean ± SEM of three independent experiments. * *p* < 0.05; ** *p* < 0.01; *** *p* < 0.001 compared to control.

The effects of the ELF-EMF on mitochondrial activity were confirmed also in this cancer model. In fact, the exposition to the specific EMF upregulated the transcript levels of proteins involved in mitochondrial respiration (Figure 5). Both the adherent and spheroid cultures were sensitive to the action of the ELF-EMF.

### 2.5. ELF-EMF Exposure Inhibited the Growth of Adherent Cells and Cell-Derived Spheroids and Increased the Mitochondrial Activity of Human Breast Cancer Models

**Figure 6:**
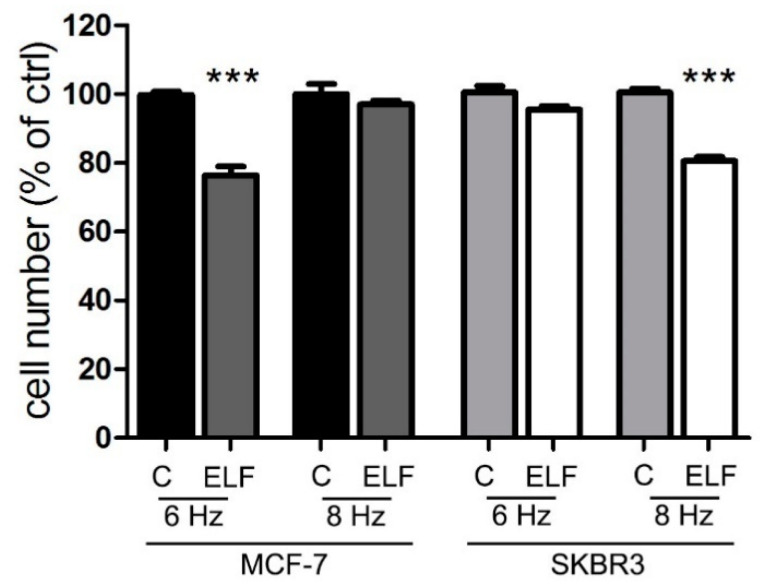
The exposure to the specific electromagnetic wave inhibits the proliferation of adherent human breast cancer cell lines. After four days of growth in standard condition (C) or in presence of ELF-EMF (ELF) at the indicated frequencies, adherent MCF-7 and SKBR3 cells were subjected to crystal violet assay. The values of the treated cells are expressed as the percentage of their respective controls (C). Data represent the means ± SEM of three independent experiments. *** *p* < 0.001 compared to the control.

To next investigate a third model of human cancer, we chose the MCF7 breast cancer cell line, since spheroids can be obtained from these cells and studied, under the appropriate conditions [40,41]. For both adherent cells and spheroids of MCF-7 cells, the calculated effective frequency was 6 Hz. As demonstrated for the other two models of pancreatic and GBM cancer, the exposure to the ELF-EMF at 6 Hz decreased the growth of adherent MCF7 cells (Figure 5). However, we could not verify the effect on spheroids because in this aggregated form, MCF7 enter into a quiescent status. Therefore, to confirm the specificity of the effect on MCF7 adherent cells, we analyzed another human breast cancer cell line, the SKBR3 cells, for which we calculated the optimal inhibitory frequency at 8 Hz. After cross-exposure, we determined that the cell lines only responded to their specific frequency, as shown in Figure 6.

**Figure 7:**
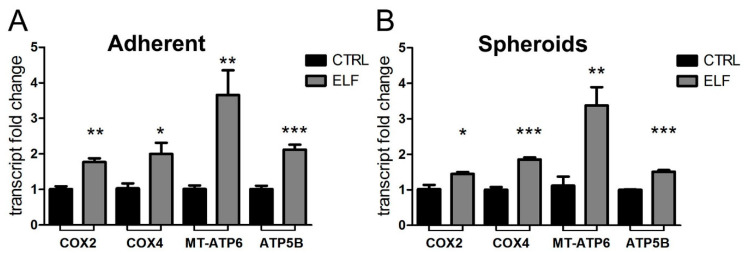
The exposure to the specific electromagnetic wave (6 Hz) enhances the expression of the respiratory chain and ATP synthase in both adherent and spheroid MCF-7 cells. Real time analysis of COX2, COX4, MT-ATP6 and ATP5B subunits transcript expression in control and in cells treated at the specific frequency of 6 Hz for both adherent cells and spheroids. Fold changes versus control are plotted on the graphs. All data represent the mean ± SEM of three independent experiments. * *p* < 0.05; ** *p* < 0.01; *** *p* < 0.001 vs. control.

Finally, also in the breast cancer model we confirmed the enhancement of mitochondrial respiration, both in adherent cells and spheroid aggregates, as shown in Figure 7A,B.

## 3. Discussion

One strategy to improve the rate of success of new cancer treatments transitioning into the clinic would be to more closely align the cellular models used in the early discovery with pre-clinical animal models and patient tumors. For solid tumors, the pre-clinical in vitro investigation can exploit the three-dimensional tumor models that more accurately recapitulate human solid tumor architecture and biology [42]. In fact, three-dimensional cell models have gained attention for their ability to mimic the features of tumors in vivo, bridging the gap between two-dimensional cell culture systems and in vivo models [43]. In line with this opportunity, we reinforced our previous novel approach based on thermodynamic analysis of cancer behavior, moving to 3D models.

For the first time, we demonstrate the efficacy of an ELF-EMF applied to 3D cancer cellular cultures. Indeed, in the present work, we compare the beneficial effects of the radiation on adherent cells and, importantly, on spheroids derived from the same solid cancer type. The scientific relevance of our results consists of: 1. The development of a novel thermodynamic analysis applied to 3D cancer models; 2. The biochemical evaluation of the impact of an ELF-EMF on 3D masses; 3. The experimental validation, which confirms the suitability of the thermodynamic approach to the more complex 3D aggregates.

We started from a consolidated and validated thermodynamic analysis on 2D cell models and we added the heat exchange analysis by considering the cells as RC circuits. This analysis leads to the conclusion that the 3D mass also responds to the thermomagnetic resonant frequency with a variation of ionic fluxes. We exploited the possibility offered by our mathematical model of calculating the thermomagnetic resonant frequency specific for each 3D model and we predicted that the generated perturbation of ion fluxes would be restored by energy expenditure detrimental to cell growth. We confirmed the validity of our thermodynamic approach both on adherent cells and on 3D structures; the latter analysis was never carried out before, to the best of our knowledge. The first evidence of efficacy was the decreased cell growth, which confirmed the validity of the theoretical model in evaluating the impact of an ELF-EMF on 3D masses and in selecting the specific resonant frequency. We then evaluated the impact on metabolism and found that mitochondrial respiration was enhanced by the selective ELF-EMF. It is of note that the increased transcription of subunits of the respiratory chain was associated with both increased levels of ATP synthase subunits and uncoupling proteins. Uncoupling proteins UCP1 and UCP2 are two widely distributed members of the anion carrier protein superfamily located in the mitochondrial inner membrane [44], which dissipate as they heat the energy contained within the mitochondrial membrane potential (Δ*ψ*_m_) through a proton leak in a process termed uncoupling [45]. The results of this study revealed that the ELF-EMF potentiated both the coupled and uncoupled respiration. We believe that, on one hand, the increased production of ATP is required and readily spent to restore the ion flux homeostasis perturbed by the exposure to the ELF-EMF. Indeed, our previous measure of ATP levels upon ELF-EMF exposure did not detect any difference [8], despite the induced respiratory activity. On the other hand, the enhanced uncoupling has the function of lowering protonic backpressure on the respiratory chain to maintain the high respiratory activity and the production of energy as ATP. Interestingly, the production of heat, as a consequence of the wasted proton gradient energy triggered by uncoupling, fits well with the thermodynamical analysis of cellular response to the ELF-EMF exposure. Indeed, we modelled cell membranes as an *RC* circuit, generating a current made of ion fluxes and dissipating heat, and we calculated the thermal resonance frequency of the ELF-EMF. The novel observation of the increased uncoupling as a response to the specific ELF-EMF nicely supports the thermodynamic biochemical model, both in 2D and 3D growth conditions.

Here, to confirm the efficacy of our approach on 3D models of deadly cancers, we chose three models of cancer characterized by aggressive behavior and survival in a hypoxic pro-metastatic environment. In fact, the formation of hypoxic areas is strongly associated with tumor growth, angiogenesis, malignant progression, metastasis and resistance to therapy [46,47].

Pancreatic ductal adenocarcinoma is characterized by a desmoplastic hypoxic stroma that plays an important role in driving progression, metastasis and chemoresistance; it is believed that hypoxia can mediate chemotherapy resistance through mechanisms, such as extrinsic resistance, regulation of drug efflux, metabolic reprogramming, alterations in apoptosis and cell survival, and induction of stemness [15].

Moreover, several studies using glioblastoma cell lines and/or clinical samples support a hypoxic up-regulation of many pro-angiogenic genes and proteins, and a hypoxia-triggered metabolic switch towards glycolysis [48]. The association of glioblastoma hypoxia with chemoresistance was also demonstrated [49].

Recognized as a hallmark of most solid tumors, hypoxia also profoundly influences multiple facets of breast cancer biology, through the Hypoxia-Inducible Factor-1 (HIF-1) mediated induction of metabolic reprogramming, neovascularization, EMT [50] and metastasis [15].

In vitro, the presence of regions of hypoxia within the spheroid core of a pancreatic tumor [51], in MCF-7 cell-derived spheroids [40,52] and within the center of glioblastoma spheroids [53], are well characterized. In this study, the analysis of transcript levels suggests that PANC-1 cells grown as spheroid aggregates downregulate the respiratory complexes, as a result of hypoxia-driven metabolic adjustment. In agreement with our observations, the hypoxic metabolic modulation has been previously demonstrated in PANC-1 spheroids [54]. Notably, as the achieved metabolic effect was evident both in the oxygenated (adherent cells) and in the hypoxic model (spheroids), we show that our thermodynamic approach can calculate the specific frequency able to enhance respiratory activity and inhibit growth even in hypoxic 3D aggregates. Further investigation is warranted to ascertain the effect of the ELF-EMF on hypoxia-driven cancer metabolism and drug resistance in these 3D cancer models.

The novelty of this study consists of the validation of the thermodynamic approach on 3D cancer models, the demonstration that the treatment with an ELF-EMF is effective in generating a mitochondrial metabolic switch and hampering the growth of 3D cancer masses also in a hypoxic environment, characterized by great resistance to drugs [55]. The clinical treatments applied to the cancers investigated in this work are far from definitive; on the basis of the results obtained on 3D models recapitulating some critical features of real tumors, the exposure to the specific ELF-EMF could be added to improve treatment outcome. This technology and the relative thermodynamic model are therefore particularly valuable and promising for future application in vivo.

## 4. Materials and Methods

### 4.1. Cell Culture

Three human cancer cell lines, representative of three different human solid tumors, were used in this study and cells were cultured both as adherent cells and spheroids, as described below.

Human pancreatic epithelioid carcinoma cell line (PANC-1) was purchased from American Type Culture Collection (ATCC, USA). Cells were cultured in a DMEM medium supplemented with 10% fetal bovine serum (FBS) and 1% antibiotics (penicillin-streptomycin) at 37 °C in humidified 5% CO_2_ atmosphere. To perform spheroids, PANC-1 cells were dissociated by trypsin and seeded in ultra-low attachment plates (Corning; Lowell, MA, USA) and in serum-free DMEM, supplemented with 10 ng/mL basic fibroblast growth factor, 20 ng/mL epidermal growth factor, and 5 μg/mL insulin [56]. Spheroids were formed after 7 days.

Primary human glioblastoma cells 010627 were obtained from surgical samples from Neurosurgical Units, Universities of Torino and Novara, after obtaining written-informed consent, and used within passage 5 [57]. The genetic background or clinical outcome of patients are listed in [57]. Cells were cultured as differentiated/adherent cells (AC) or neurospheres (NS) as previously described [58], with minor modifications [59]. For AC, Dulbecco′s modified Eagle′s medium (DMEM) supplemented with 1% *v*/*v* penicillin–streptomycin and 10% *v*/*v* FBS was used. For NS, a DMEM-F12 medium was supplemented with 1 mol/L HEPES (4-(2-hydroxyethyl)-1-piperazine ethanesulfonic acid), 0.3 mg/mL glucose, 75 mg/mL NaHCO_3_, 2 mg/mL heparin, 2 mg/mL bovine-serum albumin (BSA), 2 mmol/L progesterone, 20 ng/mL epidermal growth factor (EGF) and 10 ng/mL basic fibroblast growth factor (b-FGF). AC were obtained from dissociated NS cells, centrifuged at 1200× *g* for 5 min and seeded in AC medium. In vitro, clonogenicity and self-renewal and in vivo tumorigenicity were reported [60]. Cell phenotypic characterization is detailed in [61]. *Mycoplasma spp* contamination was assessed by PCR every 3 weeks; contaminated cells were discharged.

Human breast carcinoma MCF-7 and SKBR3 cell lines were obtained from the American Type Culture Collection (Rockville, MD, USA) and grown as a subconfluent monolayer in humidified incubator at 37 °C, 5% CO_2_ and 20% O_2_. The RPMI 1640 medium, containing 2 mM L-glutamine, 1% (*v*/*v*) antibiotic/antimycotic solution and 10% (*v*/*v*) FBS, was used as a culture medium. SKBR3 cells were used to verify the specificity of the selected electromagnetic frequencies. Spheroids were obtained after seeding MCF-7 cells in 96-well ultra-low attachment plates (Corning; Lowell, MA, USA) for 7 days.

Unless otherwise specified, reagents were purchased from Merck (Milan, Italy), whereas plastic ware was from Falcon (Becton Dickinson, Franklin Lakes, NJ, USA).

### 4.2. Cell and Spheroid Size Evaluation and ELF-EMF Exposure Treatment

Cell and spheroid size were evaluated by photographs taken in different areas of the dish. For each adherent cell type and for spheroids, cellular and nuclear sizes were measured using an ImageJ software analysis on 30 images (Sun Microsystems Inc., Palo Alto, CA, USA) and the values were used to calculate the specific frequencies. During experiments, cells were seeded in 96-multiwell plates for proliferation assays or in 6-multiwell plates for BrdU and PCR analysis, and were continuously exposed to the ELF-EMF for the required time and at the specific frequencies (14, 10, 6, 4 or 3 Hz), calculated by the thermodynamic model. The experimental setup has been previously described [8,10,62]. Briefly, the setup consists of two independent couples of coaxial coils wound into a frame of cylindrical shape, with an outer radius of 8 cm and a distance between the two coaxial coil couples of 8 cm. The experimental setup was placed inside an incubator, in order to maintain the cells living conditions. The cells were placed in the center of this apparatus and were exposed to an electromagnetic wave, generated by the device itself. The AC current signal was generated as a sine wave at the characteristic frequency and at the intensity of 70 μT. The outer coils were supplied with a DC current, and they provided a constant magnetic field of 45 μT [62]. The experimental setup was placed in the incubator inside a box made by G-iron that shielded the apparatus from the external magnetic field. The environmental earth electromagnetic field was produced by two direct current coils, while the ELF-EMF was produced by two variables currents coils.

### 4.3. Cell Proliferation Assay

The effect of the ELF-EMF on the cell growth of the different human cancer cell lines cultured both as adherent and spheroids was determined by BrdU incorporation assay and crystal violet staining, as previously reported [8], after 2 and 4 days, respectively, of incubation with or without exposure to the ELF-EMF. The BrdU assay was carried out on cells cultured for 2 days in standard conditions or in presence of ELF-EMF exposure. The incorporation of BrdU estimated the number of duplicating cells at the time of the assay; the proliferation rate was determined by the colorimetric Cell Proliferation ELISA BrdU kit (Roche Applied Science, Penzberg, Germany), following the manufacturer′s instructions. The data collected from 6 wells were averaged for each experimental condition, and each experiment was repeated three times. For crystal violet staining, at the end of 4 days exposure, cells were fixed for 15 min with 11% glutaraldehyde; plates were washed three times, air-dried and stained for 20 min with 0.1% The bound dye was solubilized with 10% acetic acid solution and the absorbance was determined at 595 nm. The crystal violet assay evaluated proliferation as the number of cells in the well (proportional to the staining). Values from 12 wells were averaged for each experimental condition and the experiment was repeated three times.

### 4.4. Reverse Transcription-Polymerase Chain Reaction (qRT-PCR)

PANC-1, MCF-7 and GBM, both as adherent cells and spheroids, were cultured for 2 days in standard conditions or exposed to the ELF-EMF at the specific frequencies. Total RNA was extracted with TRIzol (Invitrogen, Thermo Fisher Scientific, Waltham, MA, USA). Total RNA (1 µg) was reversely transcribed into cDNA and quantitative PCR was carried out as previously described [8]. Specific primers amplified the transcripts of the following human genes: cytochrome *c* oxidase subunit 2 (COX2, fwd 5′-TCTGGTCAGCCCAACTCTCT-3′, rev 5′-CCTGTGATCCACCAGAAGGT-3′), cytochrome *c* oxidase subunit 4 (COX4, fwd 5′-CGAGCAATTTCCACCTCTGT-3′, rev 5’-GGTCAGCCGATCCATATAA-3’), ATP synthase subunit beta (ATP5B, fwd 5′-GTGGGCTATCAGCCTACCCT-3′, rev 5′-CAAGTCATCAGCAGGCACAT-3′) [62], mitochondrial ATP synthase F0 subunit 6 (MT-ATP6, fwd 5′-CCAATAGCCCTGGCCGTAC-3′, rev 5′-CGCTTCCAATTAGGTGCATGA-3′), uncoupling protein 1 (UCP 1, fwd 5′-CTGGAATAGCGGCGTGCTT-3′, rev 5′-AATAACACTGGACGTCGGGC-3′) [63], uncoupling protein 2 (UCP 2, fwd 5′-GGTGGTCGGAGATACCAAA-3′, rev 5′-CTCGGGCAATGGTCTTGTAG-3′) [64] and beta 2-microglobulin (β2M, fwd 5′-AGCAAGGACTGGTCTTTCTATCTC-3′, rev 5′-ATGTCTCGATCCCACTTAACTA-3′) [8]. PCR amplification was one cycle of denaturation at 94 °C for 32 min, 45 cycles of amplification, including denaturation at 94 °C for 30 s, and annealing/extension at 60 °C for 30 s. The quantification of each sample was carried out comparing each PCR gene product with β2M, used as a reference gene to normalize the cDNA in different samples. Data were analyzed using the 2_ΔΔCT method. Analyzed transcripts exhibited high linearity amplification plots (*r* > 0.97) and similar PCR efficiency, confirming that the expression of each gene can be directly compared. The specificity of PCRs was confirmed by melt curve analysis. Nonspecific amplifications were never detected.

### 4.5. Statistical Analysis

Statistical analysis was performed using an unpaired two-tailed Student′s *t*-test or ANOVA with Tukey′s post hoc correction, where relevant. *p*-values of less than 0.05 were considered significant. All data were expressed as mean ± s.d. of three independent experiments.

## 5. Conclusions

In this study, for the first time, we demonstrated that the thermodynamic biochemical model selecting the thermomagnetic resonant frequency of the ELF-EMF specific for each cancer type can also be applied to 3D masses, in which cells exhibit complex and synergic interactions. The multicellular tumor spheroid models, representative of a hypoxic hostile microenvironment, offer new evidence that the ELF-EMF is a potential innovative strategy for treating patients with an aggressive phenotype in the future.

## Data Availability

The data presented in this study are available within the article and Supplementary Material.

## Supplementary Materials

The following supporting information can be downloaded at: https://www.mdpi.com/article/10.3390/ijms23147955/s1.

## Appendix A

In this study, for the first time, we demonstrated that the thermodynamic biochemical model selecting the thermomagnetic resonant frequency of the ELF-EMF specific for each cancer type can also be applied to 3D masses, in which cells exhibit complex and synergic interactions. The multicellular tumor spheroid models, representative of a hypoxic hostile microenvironment, offer new evidence that an ELF-EMF is a potential innovative strategy for treating patients with an aggressive phenotype in the future.

Cells are open complex thermodynamic systems, but they can be also regarded as complex engines which execute a series of chemical reactions. Energy transformations, thermo-electro-chemical processes and transports phenomena can occur across the cell’s membranes. Consequently, cells can also actively modify their behaviors in relation to changes in their environment. Moreover, all living systems waste heat, related to their internal irreversibility. This heat is outflow towards the cell environment. The analysis of irreversibility related to this wasted heat allows us to study the behavior of the cell behaviors.

We consider the living systems as black boxes and analyze only the inflows and outflows and their changes in relation to the modification of the environment. Therefore, information on the systems can be obtained by analyzing the changes in the cell heat wasted in relation to external perturbations.

Since 2012 [65,66,67,68], the fundamental quantity used in this analysis was the entropy [65,66,69,70], related to systems changes, highlighted as the only effective criterion for spontaneity of change in any system, with particular regards to the entropy variation due to irreversibility, here named entropy generation [71], which is the result of the global effect of the entropy variation

1. due to the interaction with the environment
2. within the system itself.

So, bioengineering thermodynamics is based on just two fundamental concepts of physics: interactions and flows. The result is the analytical formulation of flow-based analysis in thermodynamics, which can play the role of a “rallying point” of the different modelling approach to biosystems. Indeed, if we consider natural systems, we can highlight that they are always open systems, which means that they can exchange heat and mass with their environment. We consider the environment as a thermostat and the system, together with its environment, is an adiabatic closed system [71]. However, for an adiabatic close system, the total entropy is defined as:

(A1) $$dS=d_{i}S+d_{e}S$$

It always increases, as a consequence of the second law [71]. In relation, (A1) *dS* is the variation of the total entropy, *d_e_S* is the entropy variation for interaction between the open system considered and its environment, and *d_i_S* is the entropy variation due to irreversibility, such that:

(A2) $$\frac{dS}{dt}\geq 0$$

Now, we can write the relation (A1) as [72]:

(A3) $$\frac{dS}{dt}=\int_{V}\left[-\nabla \cdot \left(\frac{Q}{T}\right)+{\dot{s}}_{g}\right]dV$$

where Q is the heat flow, *T* is the temperature, *V* is the volume, *t* is the time and ${\dot{s}}_{g}$ is the density of the entropy generation rate. Now, we consider that the stationary states of the open system correspond to the equilibrium states of the adiabatic closed system. At stationary state entropy reaches its maximum value [73], so:

(A4) $$dS=0\;\Rightarrow \;\left[-\nabla \cdot \left(\frac{Q}{T}\right)+{\dot{s}}_{g}\right]\;=0$$

And

(A5) $$\nabla \cdot \left(\frac{Q}{T}\right)={\dot{s}}_{g}$$

This last relation allows us to state that the flows between the open system and its environment cause the entropy generation rate density, so the interaction between system and environment is responsible of irreversibility. However, we cannot state if the cause of changes is the change of the entropy inside the cell or the fluxes across the cell membrane. We can only highlight the relation between changes and fluxes, but this approach does not allow us to establish if the fluxes cause entropy changes or if entropy changes causes fluxes.

Now, considering that the entropy generation rate density can be written as [74]:

(A6) $${\dot{s}}_{g}=\sum_{k}J_{k}\cdot X_{k}$$

where **J***_k_* is the flow of the *k*-th quantity involved in the process considered and **X***_k_* is the related thermodynamic force. Now, considering that:

(A7) $$\nabla \cdot \left(\frac{Q}{T}\right)=Q\cdot \nabla \left(\frac{1}{T}\right)+\frac{1}{T}\nabla \cdot Q=\sum_{k}J_{k}\cdot X_{k}$$

The relation (A5) becomes:

(A8) $$\frac{1}{T}\nabla \cdot Q=\sum_{k}J_{k}\cdot X_{k}-Q\cdot \nabla \left(\frac{1}{T}\right)$$

This is in agreement with Le Chatelier′s principle [73], for which any change in concentration, temperature, volume or pressure generates a readjustment of the system in opposition to the effects of the applied changes in order to establish a new equilibrium, or stationary state.

Any readjustment of the state of the system can be obtained only by generating fluxes of free energy which entail any process where the system evolves from one state to another. Living cells are separated from their environment by the lipid bilayer membrane, which presents a different concentration of specific ion species on both sides. As a consequence, a charge separation across the membrane is generated by the electro-diffusion of ions down their electrochemical gradient. These ions move into a negative (inside the cell) membrane potential of around −70 to –100 mV. The hydrophobic component of the lipid bilayers behaves as a capacitor dielectric, which maintains the ionic gradients across the membrane; in some instances, the action of ATP-driven ionic pumps supports this effect by separating the charges. To understand how the fluxes are regulated across the membrane, we consider the concentration of the ions on the opposite sides of the membrane [75]:

(A9) $$c_{outside}=c_{inside}\;\exp\left(\frac{\Phi_{outside}-\Phi_{inside}}{RT}\right)$$

where *c* is the molar concentration of the chemical species, *R* is the universal constant of gas, *T* is the temperature and Φ is the electric potential energy. As a consequence of this concentration difference, the cell can move the ions and change the pH inside and outside its membrane. The ion drift velocity *v_drift_* across the cell membrane can be obtained by using the classical kinetic theory [12] as:

(A10) $$v_{drift}=\frac{Ze}{m}\;\frac{\varphi }{d}\;\tau_{drift}$$

where *Ze* is the electric charge of the ion, *m* is the ion mass, *φ* is the electric potential across the membrane, *d* is the length of the membrane and *τ**_drift_* is the mean time between two collisions [75]:

(A11) $$\tau_{drift}=\frac{m\sigma }{n{\left(Ze\right)}^{2}}$$

where *σ* is the electric conductivity. Consequently, an electric current *I* occurs for each ion *i* = H^+^, Na^+^, K^+^, Ca^2+^, Cl^−^, Mg^2+^, etc.:

(A12) $$I_{i}=n_{i}AZ_{i}ev_{drift}$$

where *A* is the mean surface area of the membrane. Now, considering the equivalent RC electric circuit for a membrane, it is possible to state that the resonant frequency for such a circuit results in (2π*RC*)^−1^, where *R* is the electric resistivity for the ion considered and *C* is the membrane capacity. It follows, that if we want to study the cross-membrane flux, we must impact the current. The easier physical way to interact with a current is to use an electromagnetic wave of the resonant frequency for the membrane, in relation to the ion considered, with its amplitude being related to the entropy generation as just obtained in Refs. [76,77,78].

Moreover, we wish to highlight that the amplitude of oscillation at the power frequency must be compared to the amplitude of oscillation of the same particle produced randomly by thermal noise, as deeply analyzed in Ref. [79]. The analysis of the signal obtained by our system has been developed in Ref. [77]: the theoretical results was confirmed by a comparison with experimental results obtained in Ref. [80]. The energy exchanged by entropy variation due to resonant thermal flux resulted of the order of 10^−19^ J, while the thermal noise can be evaluated of the order of 10^−21^ J, with the consequence that, in our experiments, no disturbance is caused by the thermal noise.
