# Supplementary figures and images for: Thermomagnetic Resonance Effect of the Extremely Low Frequency Electromagnetic Field on Three-Dimensional Cancer Models
Source: Int J Mol Sci. 2022 Jul 19;23(14):7955. doi: 10.3390/ijms23147955 (PMC9318636; doi:10.3390/ijms23147955)

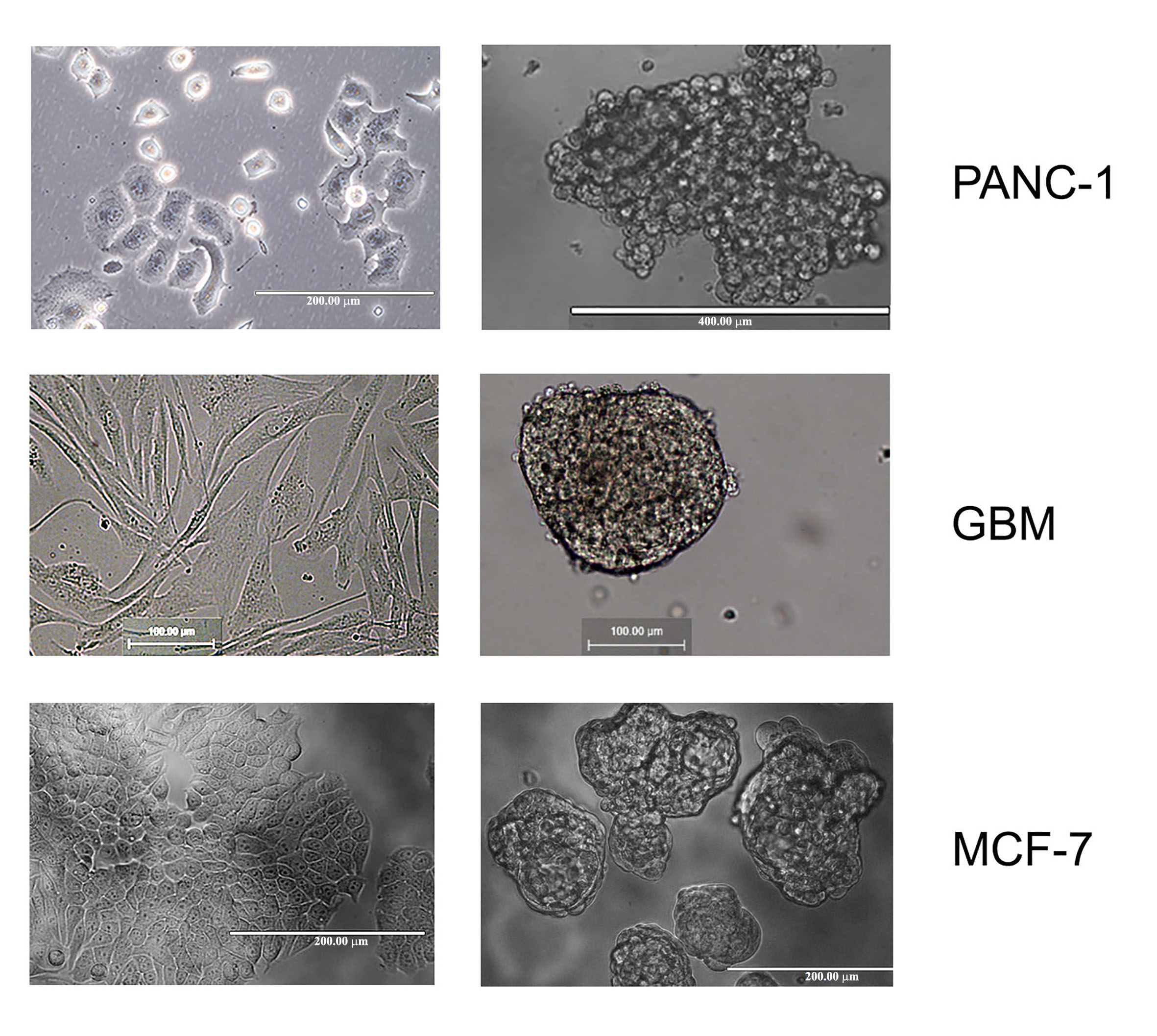

Supplement: Supplementary file 1 [file ijms-23-07955-s001.zip › Figure S1.jpg]
